# The Effect of Granulocyte Colony-Stimulating Factor (G-CSF) on Early Complications and Graft-Versus-Host Disease (GVHD) in Allogeneic Stem Cell Transplantation (ASCT) Recipients

**DOI:** 10.7759/cureus.46105

**Published:** 2023-09-28

**Authors:** Lale Aydın Kaynar, Zübeyde Nur Özkurt

**Affiliations:** 1 Adult Hematology, Gazi University, Ankara, TUR

**Keywords:** complication, gvhd, neutrophil engraftment, allogeneic stem cell transplantation, g-csf

## Abstract

Objectives

Granulocyte colony-stimulating factor (G-CSF) is commonly used to accelerate neutrophil recovery after allogeneic stem cell transplantation (ASCT) in most transplant centers. There was no consensus on the optimal use of G-CSF after ASCT. Although we use G-CSF to minimize morbidity and mortality, G-CSF can increase the risk of graft-versus-host disease (GVHD). In our study, we want to show the effect of prophylactic G-CSF on infection frequency, neutrophil and platelet engraftment, the duration of neutropenia, the development of GVHD, hospitalization time, and transplant-related mortality (TRM) after ASCT.

Materials and methods

One hundred (71 males and 29 females) patients who did not receive G-CSF and 100 (58 males and 42 females) patients who received prophylactic G-CSF were included in the study.

Results

Age, diagnosis, the time between diagnosis and transplantation, preparation regimen, donor type, and the number of infused cluster of differentiation (CD) 34+ cells were not different in both groups (p>0.05). The frequency of female patients was higher in the group receiving G-CSF. Febrile neutropenia was more frequent in patients who did not receive G-CSF. Neutrophil engraftment and platelet engraftment were detected longer in patients not receiving G-CSF. The frequency of veno-occlusive disease (VOD) and hyperacute, chronic, and acute GVHD was not different in both groups (p>0.05). One hundred-day TRM and five-year overall survival (OS) were similar in the two groups (p>0.05).

Conclusions

Our study supports that G-CSF usage does not cause an increase in the frequency of GVHD and has a positive effect on the process by accelerating myeloid engraftment. In light of the data in our study, we can say that the use of G-CSF should be investigated in a randomized controlled clinical trial.

## Introduction

Allogeneic stem cell transplantation (ASCT) is usually used in many benign and malignant hematological diseases [1]. Granulocyte colony-stimulating factor (G-CSF) is a protein, and it stimulates new blood cell growth [1]. After ASCT, G-CSF significantly accelerates the differentiation of granulocytes, so it is the standard of supportive treatment [1,2]. The administration of G-CSF to shorten neutrophil engraftment time after ASCT has become a general practice [3]. The optimal time to start G-CSF treatment is not known after transplantation [2-7]. To cluster of differentiation (CD) 34, G-CSF after ASCT is not well established, with an increased risk of graft-versus-host disease (GVHD) [8-11]. It has been suggested that the increased interleukin (IL) 6 level in donor cells when G-CSF is given after ASCT is a reason for the increased GVHD [10]. In many transplantation centers, G-CSF is used to shorten the number of neutropenic days in patients after stem cell transplantation [7]. However, there is no consensus on the standard use of G-CSF after transplantation; by adding prophylactic G-CSF to the transplantation procedure, infection-related morbidity and mortality and hospital stay can be shortened [7].

In our study, we want to show the effect of prophylactic G-CSF on infection frequency, neutrophil and platelet engraftment, the duration of neutropenia, the development of GVHD, hospital stay, and transplant-related mortality (TRM) after ASCT.

## Materials and methods

The study was approved by the Gazi University Clinical Research Ethics Committee (approval number 77082166-302.08.01, dated 10 February 2017). Two hundred patients who underwent allogeneic stem cell transplantation between February 2010 and January 2017 were retrospectively included in the study. Patients with multiple transplants and a high risk of primary rejection or graft failure and an earlier initiation of G-CSF were excluded. G-CSF prophylaxis was routinely initiated in ASCT since February 2014 in our center, so 100 consecutive patients from that date were enrolled in the G-CSF group. One group consisted of patients who underwent allogeneic stem cell transplantation in our center between 2010 and 2014 who did not receive GCSF prophylaxis. The other group consisted of the prophylaxis group who received 30 MU/day filgrastim subcutaneously for a median of 10 (3-26) days from the +5 day until the neutrophil was >1000 µ/L.

Data were analyzed with the Statistical Package for Social Sciences (SPSS) V23 (IBM SPSS Statistics, Armonk, NY). Conformity to normal distribution was examined by the Kolmogorov-Smirnov tests. The chi-square test, the Yates correction, and Fisher's exact test were used to compare categorical data according to groups. The Mann-Whitney U test was used to compare the quantitative data that were not normally distributed according to the paired groups. The log-rank (Mantel-Cox) test was used to compare the survival time. Analysis results were presented as mean±standard deviation and median (minimum-maximum) for quantitative data and frequency (percent) for categorical data. The significance level was taken as p<0.050.

## Results

One hundred (71 males and 29 females) patients who did not receive G-CSF and 100 (58 males and 42 females) patients who received G-CSF prophylaxis were included in the study. The prophylaxis group received G-CSF from the +5 day until the neutrophil was >1000 µ/L. The number of days they received G-CSF was a mean of 10.53±4.0 (minimum-maximum: 3-26) days. The mean age of the patients was found to be 39.2±14.2 (minimum-maximum, 18-67; median, 38). Eighty-two (41.0%) patients were diagnosed with acute myeloblastic leukemia (AML), 52 (26.0%) patients with acute lymphoblastic leukemia (ALL), 16 (8.0%) patients with multiple myeloma (MM), 16 (8.0%) patients with myelodysplastic syndrome (MDS), 14 (7.0%) patients with non-Hodgkin lymphoma (NHL), six (3.3%) patients with chronic myeloproliferative disease (CMPD), five (2.5%) patients with Hodgkin lymphoma (HL), five (2.5%) patients with chronic myeloid leukemia (CML), three (1.5%) patients with paroxysmal nocturnal hemoglobinuria (PNH), and one (0.5%) patient with chronic lymphocytic leukemia (CLL) in both groups. One hundred forty-five (72.5%) patients were treated with myeloablative ASCT and 55 (27.5%) patients with nonmyeloablative ASCT. The frequency of female patients was higher in the group receiving G-CSF (p=0.038). Diagnosis, donor type, and preparation regimen were not different in both groups (p>0.05). Febrile neutropenia attacks were more frequent in patients who did not receive G-CSF than those who received G-CSF (p=0.023). The frequency of veno-occlusive disease (VOD), hyperacute and acute graft-versus-host disease (GVHD), and chronic GVHD was not different in both groups (p>0.05) (Table 1).

**Table 1:** Comparison of some frequencies of patients who received and did not receive prophylactic G-CSF. *Line percentage AML, acute myeloblastic leukemia; ALL, acute lymphoblastic leukemia; MDS, myelodysplastic syndrome; MM, multiple myeloma; NHL, non-Hodgkin lymphoma; HL, Hodgkin lymphoma; CMPD, chronic myeloproliferative disease; PNH, paroxysmal nocturnal hemoglobinuria; CLL, chronic lymphocytic leukemia; CML, chronic myeloid leukemia; GVHD, graft-versus-host disease; TRM, transplant-related mortality; OS, overall survival; G-CSF, granulocyte colony-stimulating factor

Variables		Did not receive prophylactic G-CSF		Received G-CSF prophylaxis		p
		n	%*	n	%*	
Gender	Female	29	40.8	42	59.2	0.038
	Male	71	55.0	58	45.0	
Diagnosis	AML	36	43.9	46	56.1	0.062
	ALL	23	44.2	29	55.8	
	MDS	7	43.8	9	56.2	
	MM	14	87.5	2	12.5	
	NHL	7	50.0	7	50.0	
	HL	2	40.0	3	60.0	
	CMPD	4	66.7	2	33.3	
	PNH	3	100.0	0	0.0	
	CLL	1	100.0	0	0.0	
	CML	3	60.0	2	40.0	
Donor type	Relative	86	52.1	79	47.9	0.560
	Nonrelative	14	40.0	21	60.0	
Conditioning regimen	Myeloablative	75	51.7	70	48.3	0.428
	Nonmyeloablative	25	45.5	30	54.5	
Febrile neutropenia attack	Yes	86	54.1	73	45.9	0.023
Veno-occlusive disease	Yes	12	42.9	16	57.1	0.415
Hyperacute GVHD	Yes	10	58.8	7	41.2	0.447
Acute GVHD	Yes	38	52.1	35	47.9	0.659
Chronic GVHD	None	69	50.4	68	49.6	0.513
	Local	26	53.1	23	46.9	
	Extensive	5	37.7	9	64.3	
TRM (first 100 days)	Yes	11	55.0	9	45.0	1.000
OS (five years)	Yes	53	45.3	64	54.7	0.312

**Figure 1:**
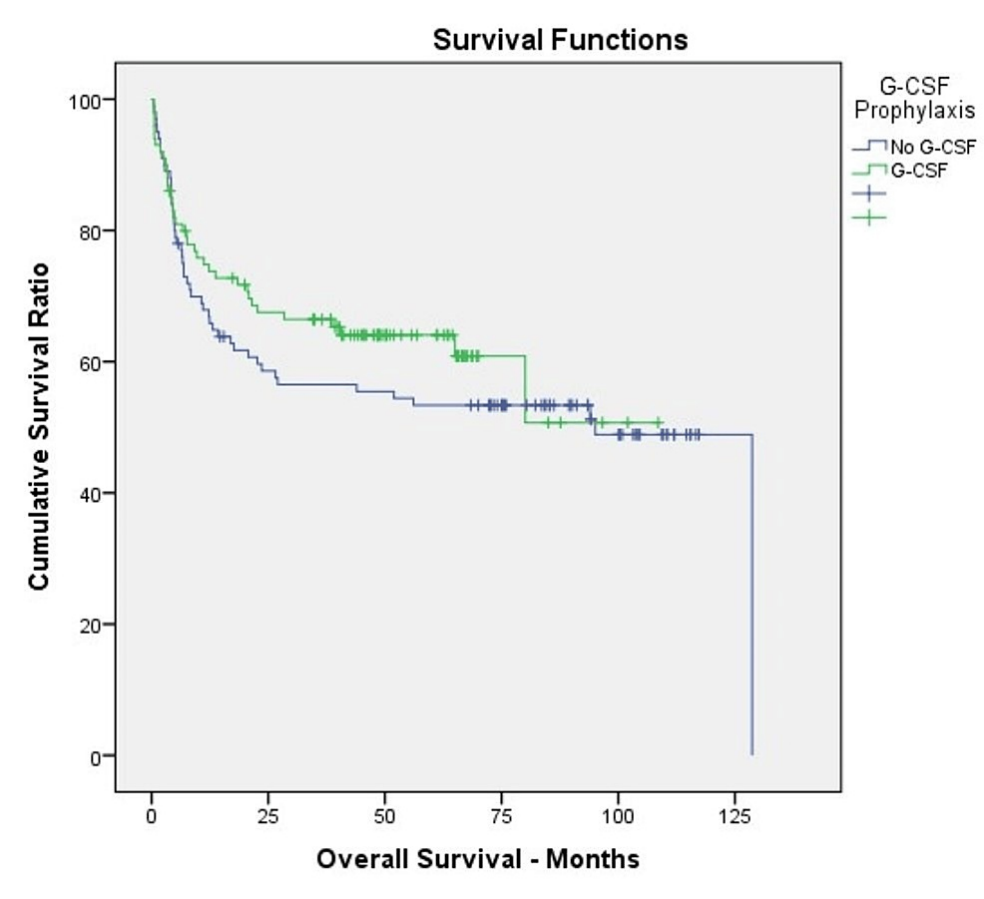
Overall survival in patients who received and did not receive prophylactic G-CSF (p=0.312). G-CSF: granulocyte colony-stimulating factor

Age, the time between diagnosis and transplant, and the number of infused CD34+ cells were not different in both groups (p>0.05). The number of days of neutropenia was similar in both groups. Neutrophil engraftment and platelet engraftment were detected later in patients not receiving G-CSF (p<0.05). Erythrocyte and platelet transfusion requirements were similar in both groups (p>0.05). Again, mean hospital stay was found to be similar in both groups (p>0.05) (Table 2). During a median follow-up of 44 (minimum-maximum: 0.4-128) months, 100-day transplant-related mortality (TRM) and five-year overall survival (OS) were similar in patients who received and did not receive prophylactic G-CSF (p>0.05) (Figure 1).

**Table 2:** Comparison of some means of patients who received and did not receive G-CSF prophylaxis. SD, standard deviation; CD34, cluster of differentiation 34; G-CSF, granulocyte colony-stimulating factor

Variables	Did not receive prophylactic G-CSF		Received G-CSF prophylaxis		p
	Mean	SD	Mean	SD	
Age	38.2	13.3	40.3	14.9	0.055
Diagnosis-transplantation time (day)	730.2	1031.3	548.8	792.7	0.443
Given CD34+ cells (×10e6/kg)	4.4	0.7	4.9	0.9	0.646
Neutropenia (day)	15.5	6.4	14.4	5.9	0.202
Neutrophil engraftment (day)	16.5	5.2	14.6	5.2	0.019
Platelet engraftment (day)	15.7	9.3	13.5	5.9	0.050
Hospital stay (day)	21.2	8.9	20.9	6.7	0.938
Erythrocyte transfusion requirements	3.7	6.7	4.3	4.7	0.136
Platelet transfusion requirements	2.2	2.3	3.2	3.7	0.051

## Discussion

Granulocyte colony-stimulating factor (G-CSF) is commonly used to accelerate neutrophil recovery after allogeneic bone marrow transplant (BMT) in most transplant centers. There was no consensus on the optimal use of G-CSF after hematopoietic stem cell transplantation (HSCT) [2,4,12]. The optimal time to start G-CSF treatment is not known [2-7]. The number of studies using G-CSF after ASCT is not that many because there are concerns about G-CSF increasing relapse and GVHD [10]. The positive role of G-CSF in hastening the myeloid recovery of patients undergoing ASCT has been established before [13,14]. However, increased levels of inflammatory cytokines and the activation of lymphocytes might increase the severity of GVHD [15,16]. Several trials, analyzing the use of G-CSF after ASCT, have shown a significant acceleration in neutrophil recovery, without an increase in the incidence and severity of GVHD, but some trials found an increase in GVDH. Whether G-CSF alone or an accelerated rate of neutrophil engraftment triggers or potentiates acute GVHD is unclear [17].

Remberger et al. showed that the neutrophil engraftment time was significantly shortened in patients receiving G-CSF. No significant difference was observed in the platelet engraftment time. There was no difference between erythrocyte and granulocyte transfusion numbers and infection rates. The number of platelet transfusions was less in the group receiving G-CSF. However, the incidence of GVHD was found to be higher in patients given G-CSF prophylaxis than in the other group [17]. Serum soluble interleukin receptor levels were higher in patients who developed acute GVHD during G-CSF administration than in those who developed acute GVHD after it had been stopped. This finding suggests that the administration of G-CSF may aggravate acute GVHD [17]. In our study, we showed similar results on neutrophil engraftment and erythrocyte transfusions except for GVHD results and platelet engraftment.

Sing et al. showed that neutrophil engraftment was earlier and posttransplant hospital stay was shorter in patients receiving G-CSF [3]. A higher rate of extensive chronic GVHD was found in patients using G-CSF [3]. In their study, Ringdén et al. showed that neutrophil engraftment was earlier and platelet engraftment was longer in the group receiving G-CSF [11]. At the same time, in this study, G-CSF was found to be associated with an increase in acute and chronic GVHD and an increase in TRM [11]. In our study, we found earlier neutrophil engraftment, but there was no significant difference in hospital stay and the frequencies of GVHD.

Dekker et al. (2006) found that after ASCT, G-CSF reduced the risk of documented infections and the duration of parenteral antibiotics but did not reduce infection-related mortality in their systematic review and meta-analysis [18]. In another meta-analysis, Gupta et al. showed that neutrophil engraftment occurred earlier in patients using G-CSF [19]. They found no difference in the platelet engraftment time between the two arms, and there was no difference in terms of length of hospital stay and OS [19].

Several studies have shown an increased incidence of GVHD with the use of G-CSF. In their study, Trivedi et al. found that the administration of G-CSF to patients was not associated with an increase in the incidence of GVHD [2]. In a meta-analysis, Gupta et al. showed no increase in acute or chronic GVHD with G-CSF and no difference in the duration of hospital stay post HSCT [19]. Ho et al. showed in their meta-analysis no significant effect of the G-CSF on the risks of GVHD or early TRM in the heterogeneous group [10].

In our study, not significant difference was found between the two groups in acute and chronic GVHD.

There are several limitations in our study. It has a non-randomized retrospective design, and the control group consisted of patients who underwent allogeneic stem cell transplantation in our center between 2010 and 2014 who did not receive GCSF prophylaxis (mentioned as a historical group).

## Conclusions

Our study supports that G-CSF usage does not cause an increase in the frequency of GVHD and has a positive effect on the process by accelerating myeloid engraftment. The reduction of febrile neutropenia attacks with G-CSF may shorten the duration of hospital stay and contribute to reducing early mortality. In light of the data in our study, we can say that the use of G-CSF should be investigated in a randomized controlled clinical trial. At the same time, further studies are needed to determine the optimal starting time for G-CSF after ASCT.
